# Biological Roles of Aberrantly Expressed Glycosphingolipids and Related Enzymes in Human Cancer Development and Progression

**DOI:** 10.3389/fphys.2018.00466

**Published:** 2018-05-03

**Authors:** Dinghao Zhuo, Xiang Li, Feng Guan

**Affiliations:** ^1^Key Laboratory of Carbohydrate Chemistry and Biotechnology, Ministry of Education, School of Biotechnology, Jiangnan University, Wuxi, China; ^2^Key Laboratory of Resource Biology and Biotechnology in Western China, Ministry of China, College of Life Science, Northwest University, Xi’an, China

**Keywords:** glycosphingolipids, cellular processes, signaling pathway, aberrant expression, cancer development

## Abstract

Glycosphingolipids (GSLs), which consist of a hydrophobic ceramide backbone and a hydrophilic carbohydrate residue, are an important type of glycolipid expressed in surface membranes of all animal cells. GSLs play essential roles in maintenance of plasma membrane stability, in regulation of numerous cellular processes (including adhesion, proliferation, apoptosis, and recognition), and in modulation of signal transduction pathways. GSLs have traditionally been classified as ganglio-series, lacto-series, or globo-series on the basis of their diverse types of oligosaccharide chains. Structures and functions of specific GSLs are also determined by their oligosaccharide chains. Different cells and tissues show differential expression of GSLs, and changes in structures of GSL glycan moieties occur during development of numerous types of human cancer. Association of GSLs and/or related enzymes with initiation and progression of cancer has been documented in 100s of studies, and many such GSLs are useful markers or targets for cancer diagnosis or therapy. In this review, we summarize (i) recent studies on aberrant expression and distribution of GSLs in common human cancers (breast, lung, colorectal, melanoma, prostate, ovarian, leukemia, renal, bladder, gastric); (ii) biological functions of specific GSLs in these cancers.

## Introduction

**FIGURE 1:**
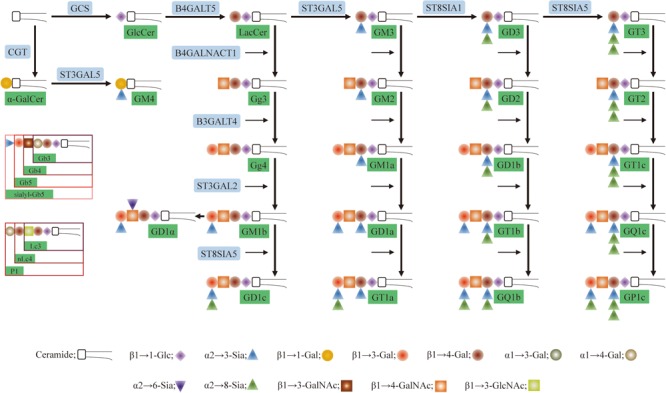
Pathway for glycosphingolipids (GSLs) biosynthesis. Glc, glucose; Lac, lactosyl; Gal, galactose; GalNAc, *N*-acetylgalactosamine; GlcNAc, *N*-acetylglucosamine; Sia, sialic acid; b, globo-series; Lc3, lactotriaosylceramide; Lc4, neolactotetraosylceramide. The code names of gangliosides are according to Svennerholm (1980). G refers to ganglioside, the second letter refers to the number of sialic acids residues (M, mono-; D, di-; T, tri-; Q, quad-; P, pent-), and the number (1, 2, 3, 4) refers to the order of migration of the ganglioside on thin-layer chromatography (e.g., GM4 > GM3 > GM2 > GM1).

Glycosphingolipids (GSLs) are a subtype of glycolipids found in all animal cell surface membranes. GSLs have three basic components: sphingosine, fatty acid, and a carbohydrate residue (Hakomori, 2002). Major structural and functional classifications of GSLs have traditionally been based on glycans. GSLs can be subclassified as neutral, sialylated, or basic, or as ganglio-series, lacto-series, or globo-series (Hakomori, 2003). Some common types of GSLs (mainly ganglio-series) and related enzymes are shown in **Figure 1**. Two important functions of GSLs are mediation of cell–cell interactions and modulation of signal transduction pathways. Many studies have focused on the role of certain GSLs as cancer biomarkers and their application in cancer immunotherapy. Differential expression profiles of GSLs associated with oncogenic transformation were first reported almost 50 years ago (Hakomori and Murakami, 1968). A steadily increasing number of subsequent studies described aberrant expression and function of GSLs and related enzymes in cancer cells and tissues. Here, we review recent studies along this line; these studies are summarized in **Table 1**.

**Table 1:** Glycosphingolipids and related enzymes aberrantly expressed in various types of cancer.

Cancer	Upregulation or promotion	Downregulation or inhibition
Breast	**GD2** and/or **GD3** (Cazet et al., 2010, 2012; Battula et al., 2012; Liang et al., 2013; Lin et al., 2014; Sarkar et al., 2015) **ST8SIA1** (Ruckhaberle et al., 2009; Liang et al., 2017) **Gb5** (Cheung et al., 2016) **GD1α** (Bos et al., 2009; Vandermeersch et al., 2015; Drolez et al., 2016)	**GD1b** (Ha et al., 2016) **Gg4** and **B3GALT4** (Guan et al., 2009, 2010; Guo et al., 2015)

Lung	**Gb3** (Tyler et al., 2015) **GM2** (Yamada et al., 2011) **NeuGcGM3** (Hayashi et al., 2013; Alfonso et al., 2014; Piperno et al., 2015; Palomo et al., 2016)	**α-GalCer** (Hasegawa et al., 2014; Ando et al., 2015; Ito et al., 2015; Yamashita et al., 2016) **GALC** (Peng et al., 2015)

Colorectal	**Gb3** (Distler et al., 2009) **Gb4** (Park et al., 2012) **GCS** (Haynes et al., 2012) **NEU3** (Shiozaki et al., 2009; Yamaguchi et al., 2012; Mozzi et al., 2015; Takahashi et al., 2015)	**GD1a** and **GM1** (Kwak et al., 2011) **α-GalCer** (Yoshioka et al., 2012; Dong et al., 2016) **GM3** (Chung et al., 2014)

Melanoma	**NeuGcGM3** (Tringali et al., 2014) **d-GM3** (Yan et al., 2013) **GD2** and **GD3** (Furukawa et al., 2014; Dobrenkov et al., 2016; Gargett et al., 2016; Kaneko et al., 2016; Makino et al., 2016) **B4GalT5** (Shirane et al., 2014)	**α-GalCer** (Neumann et al., 2015; Albertini et al., 2016)

Leukemia	**NeuGcGM3** (Fernandez-Marrero et al., 2011; Casadesus et al., 2013) **GCS** (Watters et al., 2013; Wang et al., 2014) **Lc3, GM3**, and **nLc4** (Wang et al., 2012)	**α-GalCer** (Weinkove et al., 2013) **GM3** (Jin et al., 2014; Delannoy et al., 2017) **GlcCer** (Schwamb et al., 2012)

Prostate	**GD1a** and **SPG** (Hatano et al., 2011, 2012) **sialyl-Gb5** (Sivasubramaniyan et al., 2015; Hofner et al., 2016) **LacCer** (Skotland et al., 2017) **Gg4** (Van Slambrouck et al., 2009, 2014)	**DSGb5** (Shimada et al., 2014)

Ovarian	**P1** (Jacob et al., 2014) **GD3** (Webb et al., 2012)	**GM3** (Prinetti et al., 2011)

Renal	**GM3** (Lin et al., 2012) **DSGb5** (Kawasaki et al., 2015) **LacCer** (Chatterjee et al., 2013)	**GlcCer** (Chatterjee et al., 2013)

Bladder	**GCS** (Sun et al., 2012)	**GM3** (Wang et al., 2013)

Gastric	**Gb3** (Geyer et al., 2016)	

## Breast Cancer

Breast cancer is the most common type of cancer in women (Siegel et al., 2017b). Expression of certain GSLs in breast cancer tissue are distinct from that in normal breast tissue. Gangliosides GD3, 9-*O*-acetyl-GD3, and 9*-O*-acetyl-GT3 are barely detectable in normal breast tissues, but were found to be overexpressed in ∼50% of invasive ductal carcinomas (Marquina et al., 1996). In breast phyllodes tumors, mammosphere formation capacity was 3.9-fold greater in GD2^+^ cells than in GD2^-^ cells, and the GD2^+^ subpopulation displayed more mesenchymal stem cell characteristics (Lin et al., 2014). GD3/GD2 synthase *ST8SIA1* was overexpressed in estrogen receptor (ER)-negative breast cancer tumors (Ruckhaberle et al., 2009), resulting in accumulation of GD2 (Cazet et al., 2012). Such accumulation enhanced proliferation and tumorigenicity of MDA-MB-231 breast cancer cells through ganglioside-mediated activation of c-Met receptor (Cazet et al., 2010, 2012; Sarkar et al., 2015). GD2 was identified as a specific cell surface marker of CD44^hi^/CD24^lo^ breast cancer stem cells (CSCs) (Battula et al., 2012). GD2 and GD3 levels were dramatically higher in breast CSCs than in non-CSCs, and knockdown of their synthases *B4GALNT1* and *ST8SIA1* resulted in phenotypic change from CSC to non-CSC (Liang et al., 2013). Follow-up studies demonstrated that *ST8SIA1* maintains stem cell phenotype in breast CSCs, and that GD3 synthases may be involved in gefitinib-resistance of epidermal growth factor receptor (EGFR)-positive breast cancer cells (Liang et al., 2017). Stage-specific embryonic antigen (SSEA)-3, also known as Gb5, is another potential marker of breast CSCs (Cheung et al., 2016).

GD2 can be further converted to disialoganglioside GD1b. Exogenous or endogenous expression of GD1b (but not GD2) in human breast cancer MCF-7 results in apoptosis (Ha et al., 2016). Overexpression of GD1α or its synthase *ST6GALNAC5* in breast cancer cells promotes their metastasis to brain by enhancing adhesion to brain endothelial cells and reducing interactions with the blood–brain barrier (Bos et al., 2009; Vandermeersch et al., 2015; Drolez et al., 2016).

The epithelial–mesenchymal transition (EMT) phenomenon plays an important role in cancer metastasis. In normal murine mammary gland (NMuMG) cells, levels of Gg4 and its synthase *B3GALT4* were significantly reduced during transforming growth factor-β (TGF-β)-induced EMT, and exogenous addition of Gg4 suppressed TGF-β-induced changes of morphology, motility, and levels of epithelial and mesenchymal markers (Guan et al., 2009). Gg4 appears to maintain epithelial cell membrane organization through its interaction with epithelial molecules such as E-cadherin and β-catenin (Guan et al., 2010). A TGF-β signal pathway-related complex formed by transcriptional factors Smad3 and Smad4 may directly bind to *B3GALT4* promoter and reduce Gg4 expression during EMT (Guo et al., 2015).

## Lung Cancer

Lung cancer is a common cancer in both men and women, and the leading cause of cancer-related mortality (Jemal et al., 2011; Siegel et al., 2017b). Recent studies demonstrate the important roles of GSLs in lung cancer transformation and progression. α-galactosylceramide (α-GalCer) (including allogeneic sources), a specific ligand of invariant natural killer T (iNKT) cells, exerts an anti-tumor effect by increasing production of the tumor growth suppressor IFN-γ (Hasegawa et al., 2014). Several groups have attempted to enhance the therapeutic effects of α-GalCer on lung cancer. Therapeutic efficiency of α-GalCer was enhanced by inhibition of inducible nitric oxide synthase (iNOS) expression (Ito et al., 2015). Combination therapy with α-GalCer and lipopolysaccharide obviously promoted tumor antigen-specific immune responses and suppressed tumor growth (Ando et al., 2015). Host CD40 apparently plays an essential role in the effectiveness of α-GalCer treatment on lung metastasis (Yamashita et al., 2016).

Cisplatin is a chemotherapeutic agent widely used in treatment of many types of cancer. It induces cell apoptosis by increasing DNA fragmentation, inhibiting cell proliferation and activating mitochondria-dependent apoptotic pathway. Increased cell surface Gb3 expression led to acquisition of cisplatin resistance in non-small cell lung cancer (NSCLC) cells, and reduced glucosylceramide (GlcCer) synthase (GCS)-potentiated cisplatin cytotoxicity in NSCLC H1299 cells. GCS-induced Gb3 expression has a regulatory role in acquisition of cisplatin resistance in NSCLC cells (Tyler et al., 2015). Expression of galactocerebrosidase (GALC), an enzyme that removes galactose from GSLs, is reduced in lung cancer and other human cancers. Downregulation of *GALC* gene resulted from hypermethylation of its promoter, suggesting that lung cancer tumorigenesis is due in part to epigenetic inactivation of GALC (Peng et al., 2015).

N-acetylated ganglioside NeuAcGM3 is usually present in normal human tissues, whereas many human tumors express N-glycolylated ganglioside NeuGcGM3. NeuGcGM3 was present in 86 of 93 (93.5%) NSCLC samples, as shown by immunohistochemical staining (Hayashi et al., 2013). NeuGcGM3, because of its selective expression in tumors, is a potentially useful target for immunotherapy, e.g., using Racotumomab-alum vaccine (Alfonso et al., 2014) or recombinant monoclonal antibody 14F7 (Piperno et al., 2015). In cases in which NeuGcGM3 and EGFR are involved jointly in tumor cell metastasis, therapeutic strategies that simultaneously target both molecules may be effective (Palomo et al., 2016).

Ganglioside GM2 is involved in cell adhesion and cell metastasis. GM2-expressing small cell lung cancer (SCLC) cells underwent multiple organ metastases in a SCID mouse model, and these metastases were inhibited by treatment with humanized anti-GM2 antibodies BIW-8962 and KM8927 (Yamada et al., 2011).

## Colorectal Cancer

Another common cancer worldwide is colorectal cancer (Siegel et al., 2017a). Certain GSLs and related enzymes are aberrantly expressed in colorectal cancer. The glycosylation modification of GSLs during colorectal cancer progression were obtained in 13 colorectal tumor tissues, and these were found to be characterized by increased fucosylation, decreased acetylation and sulfation, reduced expression of globo-type glycans and disialyl gangliosides (Holst et al., 2013). In a study by Distler et al. (2009), 13 of 16 (81.3%) colon cancer patients showed elevated expression of the GSL Gb3 (also known as CD77). As Gb3 is the receptor of Shiga toxin and binds to the STx B-subunit or its derivatives, which are therefore potential targets for colorectal cancer treatment. Gb4, synthesized from Gb3, has been characterized as an SSEA and is highly expressed in many types of cancer. In human colorectal carcinoma HCT116 cells, Gb4 enhanced activation of EGFR-induced MAPK/ERK signaling through direct interaction with EGFR (Park et al., 2012). Human colorectal carcinoma-associated GA733 antigen, also termed epithelial cell adhesion molecule (EpCAM), is selectively expressed in human colorectal carcinoma. Expression of gangliosides GD1a and GM1 greatly enhanced the anticancer effect of anti-EpCAM mAb in human colon adenocarcinoma SW620 cells (Kwak et al., 2011).

A therapeutic effect on colorectal cancer has also been demonstrated for α-GalCer. Treatment with α-GalCer significantly reduced the number of colorectal tumors in AOM/DSS mice (Yoshioka et al., 2012). Combined treatment with α-GalCer-loaded tumor cells and cytosine-phosphorothioate-guanine (a TLR9 agonist) in a mouse colorectal cancer model led to tumor growth inhibition and prolonged survival (Dong et al., 2016). In p53-deficient HCT116 cells, GCS level was reduced by treatment with mitomycin C, a DNA-damaging agent. Apoptosis was significantly enhanced by simultaneous GCS inhibition and mitomycin C treatment in p53-deficient cells, but not in p53-expressing cells (Haynes et al., 2012). Cisplatin is also used for chemotherapy of colorectal cancer. GM3-mediated oxidative apoptosis was shown to be related to cisplatin-induced apoptosis of HCT116 cells (Chung et al., 2014).

NEU3, a human plasma membrane-associated sialidase that specifically hydrolyzes sialic acids on gangliosides, is upregulated in colorectal cancer and plays an important role in malignancy (Shiozaki et al., 2009). In a mouse model of colitis-associated colon carcinogenesis induced by azoxymethane and dextran sodium sulfate, NEU3-deficient mice were less susceptible than wild-type mice (Yamaguchi et al., 2012). NEU3 therefore seems to be involved in inflammation-dependent tumor development. NEU3 also enhances EGFR activation through desialylation without affecting EGFR mRNA or protein expression (Mozzi et al., 2015). In HT-29 and HCT116 colorectal cancer cells, NEU3 silencing significantly reduced clonogenicity and downregulated stemness and Wnt-related genes, suggesting that Wnt signaling contributes to NEU3-induced tumorigenesis through maintenance of stem-like characteristics of these cells (Takahashi et al., 2015).

## Melanoma

Melanoma is the type of skin cancer with highest mortality rate, resulting annually in ∼60,000 deaths in ∼3 million patients worldwide (Wang et al., 2016). Certain GSLs were found to be specifically expressed and serve as distinctive molecular markers in melanoma cells (Hakomori, 2001). Among patients with various types of melanoma, survival was lowest for those having high levels of GM3 (mainly NeuGcGM3) in isolated melanoma cells (“cluster 1”). Such cluster 1 cells displayed highest malignant properties in terms of growth in soft agar, *in vitro* invasiveness, and expression of anti-apoptotic proteins (Tringali et al., 2014). The deacetylated GM3 (d-GM3) variant was found in metastatic melanomas but not in non-invasive melanomas or benign nevi. d-GM3 apparently promoted metastasis of human melanoma cells via the uPAR/integrin and p38 MAPK pathways (Yan et al., 2013).

GD2 and GD3 are highly and specifically expressed in most human melanoma tissues, and their expression is correlated with malignant properties such as cell proliferation and invasiveness (Dobrenkov et al., 2016). Molecules involved in GD3-mediated signaling pathways, such as p130Cas and paxillin, are potential targets for melanoma treatment. RNAi blocking of p130Cas and/or paxillin strongly suppressed melanoma growth (Makino et al., 2016). Stimulation by hepatocyte growth factor (HGF) or adhesion to collagen type I enhanced cell proliferation and apoptosis resistance via MAPK and Akt signaling pathways in GD3+, but not GD3-, human melanoma N1 cells. Increased GD3 expression promoted melanoma cell adhesion to surrounding tissues and susceptibility to HGF present in the tumor microenvironment, leading to synergy of multiple extracellular signals in melanoma tissue (Furukawa et al., 2014). Kaneko et al. (2016) showed that enhancement of melanoma malignant properties by GD3 may result in part from recruitment of γ-secretase to rafts, facilitating efficient cleavage of neogenin. GD2-specific chimeric antigen receptor (CAR) T-cells provide another promising new approach for melanoma cancer immunotherapy. These T-cells had a strong, rapid effect on metastatic melanoma, and blocking of PD-1 promoted CAR T-cell survival and killing of PD-L1^+^ tumor cells (Gargett et al., 2016).

The lactosylceramide (LacCer) synthase B4GalT5 was upregulated during malignant transformation of mouse melanoma B16-F10 cells, and reduced expression of the *B4GalT5* gene significantly reduced tumorigenic and metastatic potential (Shirane et al., 2014). α-GalCer exerted anti-tumor effects through eff activation of iNKT cells in melanoma as in some other types of cancer (Neumann et al., 2015; Albertini et al., 2016).

## Leukemia

Leukemia is a group of cancers that usually begin in the bone marrow and lead to high numbers of abnormal white blood cells. ∼90% of leukemias occur in adults, in whom the most common types are acute myeloid leukemia (AML) and chronic lymphocytic leukemia (CLL). It is also the most common cancer in children, in whom ∼75% of cases are acute lymphoblastic leukemia (ALL). Differentiation induction therapy of leukemia has received considerable research attention. Human chronic myelogenous leukemia (CML) K562 cells induced by caffeic acid phenethyl ester (CAPE) to differentiate toward megakaryocytic lineage showed increased GM3 synthase transcriptional activity and GM3 levels (Jin et al., 2014). GM3 expression was upregulated during differentiation of human acute monocytic leukemia THP-1 cells into macrophages (Delannoy et al., 2017). However, according to Wang et al. (2012), the study found that the levels of lactotriaosylceramide (Lc3), GM3 and neolactotetraosylceramide (nLc4) are higher in AML patients bone marrow than in healthy controls, especially the M1 subtype of AML. These results may indicate that the expression of GM3 is closely related to the various leukemia subtypes. Human normal tissues lack NeuGcGM3 because of a deletion in the *cmah* gene that encodes the enzyme responsible for NeuGc synthesis. Silencing of *cmah* in NeuGcGM3-expressing L1210 mouse lymphocytic leukemia B cells suppressed the cytotoxic effect by antibody 14F7 (Fernandez-Marrero et al., 2011). In a follow-up study, these *cmah*-silenced L1210 cells displayed enhanced NeuAcGM3 expression and an inhibitory effect on anchorage-independent cell growth and tumor development *in vivo* (Casadesus et al., 2013).

In patients with early-stage CLL, iNKT cells and the CD1d axis were fundamentally intact, and treatment with α-GalCer was feasible and effective (Weinkove et al., 2013). Growth and survival of CLL cells were promoted by stimulation with B-cell receptor (BCR), CD40 ligand (CD40L), or interleukin-4 (IL-4), through regulation of apoptosis resistance. Schwamb et al. (2012) found that anti-apoptotic effect of GlcCer was significantly enhanced by BCR stimulation in primary CLL cells.

Combination treatment of leukemic NK cells with C6-ceramide nanoliposomes and PPMP (a GCS inhibitor) induced apoptosis through the intrinsic mitochondrial cell death pathway (Watters et al., 2013). In K562/A02, a multidrug-resistant variant of CML K562 cells with GCS and Bcl-2 co-overexpression, apoptosis was enhanced by adriamycin (a chemotherapeutic agent used for treatment of various cancers) through downregulation of Bcl-2 via the ERK pathway. GCS inhibition also suppressed Bcl-2 in these cells. Thus, GCS may promote apoptosis resistance via upregulation of Bcl-2 expression (Wang et al., 2014).

## Prostate and Ovarian Cancer

Prostate cancer is the second most common type of cancer in men (Ferlay et al., 2015). GD1a and sialylparagloboside (SPG) showed higher expression in castration-resistant prostate cancer PC3 and DU145 cells than in hormone-sensitive prostate cancer cells or normal prostate epithelium. Such expression was indirectly controlled by NF-κB (mainly RelB) through transcriptional regulation of GD1a and SPG synthases *ST3Gal2* and *ST3Gal6* (Hatano et al., 2011). *ST3Gal2* expression was regulated by androgen-dependent demethylation of CpG sites in its promoter (Hatano et al., 2012).

SSEA-4 (also known as sialyl-Gb5) plays an important role in prostate cancer development by affecting adhesion of cells to extracellular matrix (Sivasubramaniyan et al., 2015) and facilitating precise recognition of basal epithelial stem cell/progenitor cell lineages (Hofner et al., 2016). Disialosyl globopentaosylceramide (DSGb5) is expressed in benign prostate tissue but not in prostate cancer. However, cancer cells of patients with a worse prognosis show high expression levels of DSGb5, this alteration may indicate the progression of malignant potential of prostate cancer. Therefore, DSGb5 has a potential as a novel prostate cancer marker (Shimada et al., 2014).

Levels of LacCer (d18:1/16:0) in urinary exosomes were ∼95% higher in prostate cancer patients than in healthy controls (Skotland et al., 2017). The carbohydrate moiety of Gg4 interacts with α2,3-linked sialic acid residues of integrin α2β1, and adhesion, migration, and invasiveness of prostate cancer C4-2B cells are affected by colocalization of these molecules (Van Slambrouck et al., 2009, 2014).

Ovarian cancer is the seventh most common cancer and eighth most common cause of cancer-related death in women. Rajanayake et al. (2016) compared GSL profiles in epithelial ovarian cancer SKOV3 cells vs. benign ovarian T29 cells. Five neutral GSLs were found only in SKOV3 cells, and several sialylated GSLs were differentially expressed. Gangliosides are concentrated primarily in lipid rafts – particularly caveolae, a type of lipid raft enriched in caveolins and functioning in signal transduction. Recombinant human ovarian cancer A2780 cells overexpressing GM3 synthase displayed reduced motility due to inactivation of c-Src by ganglioside/caveolin-1 complex (Prinetti et al., 2011). The neolacto-series GSL P1 was found to be expressed on both erythrocytes and ovarian cancer cells, and appears to be a novel tumor-associated antigen associated with cell migration (Jacob et al., 2014). Webb et al. (2012) showed that GD3 in ovarian cancer ascites fluid is involved in a mechanism of early tumor immune evasion, based on its high affinity for CD1d and consequent blocking of innate immune activation of NKT cells.

## Renal, Bladder, and Gastric Cancers

Bladder, renal, and gastric cancers are also fairly common in humans. These types of cancer also display abnormal expression of certain GSLs and related enzymes.

Following implantation of renal cancer cells into BALB/c mice, increased tumor volume was accompanied by upregulation of LacCer. Tumor volume was strongly reduced by treatment with D-PDMP, an inhibitor of GCS and LacCer synthase. D-PDMP treatment led to increased GlcCer level, possibly because of reduced activity of GlcCer glucosidase (Chatterjee et al., 2013). Confusingly, in some cases a particular GSL may display opposite effects in different cancer types (see **Table 1**). For example, GM3 acts as negative regulator of most cancers, the expression of GM3 (d18:1/22:1) in renal cancer patients were higher than in healthy controls (Lin et al., 2012). Similarly, high DSGb5 expression levels exhibit greater migration potential in renal cell carcinoma cells (Kawasaki et al., 2015), but the expression of DSGb5 is decreased in the early stage of transformation of prostate cancer from benign glands. These results may suggest that certain GSLs play a very complex role during the development and progression of renal cancer.

GCS is highly expressed in bladder cancer, and correlated with poor prognosis (Sun et al., 2012). In human bladder cancer YTS-1, T24, 5637, and KK47 cells, exogenous addition of GM3 reduced cell proliferation, cell adhesion, and EGFR phosphorylation (Wang et al., 2013).

Geyer et al. (2016) identified Gb3 in gastric carcinoma patients and cell lines using recombinant variant STxB-Cy3. Immunofluorescence analysis revealed expression of Gb3 in the majority of patients (36/50; 72%) and cell lines (6/10; 60%).

## Summary and Perspectives

Glycosphingolipids play an essential role in maintaining normal physiological functions of cells. In many types of human cancer, aberrant expression of specific GSLs and related enzymes is strongly associated with tumor initiation and malignant transformation. Cancer immunotherapy is a highly promising approach that use of the immune system to treat cancer (Couzin-Frankel, 2013). In view of the aberrant expression of specific GSLs in many cancer, certain GSLs are selected as tumor-associated antigens and their antibodies are currently under preclinical studies or clinical investigation, also including molecular vaccines. For example, the antibody hu14.18K322A, which specifically recognize GD2, is being investigated in a phase II trial in neuroblastoma patients (Furman et al., 2017). Another example, the antibody BIW-8962, targets GM2, which is highly expressed in lung cancer (Lee et al., 2017). Racotumomab as an anti-idiotypic antibody vaccine that response against NeuGcGM3 can significantly extend the life of lung cancer patients by inhibiting the growth of their tumors. After a successful phase II/III study, Racotumomab adjuvanted with aluminum hydroxide was conditionally approved in Latin American countries as maintenance therapy for NSCLC (Gabri et al., 2016). Research shows NeuGcGM3 was highly expressed in many different human cancers, heralding a huge potential of Racotumomab or other NeuGcGM3-based vaccines for cancer immunotherapy. The Globo-series are another attractive targets. The antibodies against Gb3, Gb4, and Globo H have been shown to be effective as anti-tumor agents. In addition, their relevant vaccines are equally valid approach for cancer immunotherapy (Danishefsky et al., 2015). Meanwhile, certain GSLs can be used as tumor inhibitor. One example, α-GalCer exhibits a strong anti-tumor effect and new therapeutic method are undergoing clinical trials (Gasser et al., 2018). With the deepening and development of research, we believe more GSLs will be found to have tremendous applicable value on cancer therapy.

The relationships between expression of many GSL species and development of various types of cancer remain unclear. The molecular mechanisms underlying the effects of GSLs on cancer development and progression also need to be elucidated. There are more than 400 species of GSLs in mammals (Hakomori, 2003). Determine the expression of these various GSLs and understanding the functional significance of this diversity in cancer is thus a challenging task. In recent years, mass spectrometry and its correlative technology has been widely explored for the identification and quantification of GSLs due to its high resolution, sensitivity, and accuracy. But owing to the complexity of GSLs, acquisition and accurate analysis of the category of glycosidic bond and the specific oligosaccharide structures, for instance, isomers with different glycan chains such as GM1a and GM1b, same glycan chain but different ceramide portions, is still in a difficult situation (Sarbu et al., 2016). Speaking of that, the emerging need for developing the effective technology to analyze the GSL is thus requested.

Great progress has been made during the past two decades in use of certain GSLs as targets for cancer immunotherapy and diagnosis. The foreseeable progress of the glycobiology field, with the rapid expansion of new ideas and new methods, especially the improvement of mass spectrometry technology, which are providing increasing advances in the understanding of how GSLs impact cancer progression, will allow the development of a relatively unexploited field of cancer treatments based on aberrant expression of GSLs, leading to exciting and novel clinical applications.

## Author Contributions

FG conceived and designed the article frame. DZ, XL, and FG wrote the paper.

## Conflict of Interest Statement

The authors declare that the research was conducted in the absence of any commercial or financial relationships that could be construed as a potential conflict of interest. The handling Editor is currently co-organizing a Research Topic with one of the authors FG, and confirms the absence of any other collaboration.
